# Influences of exocrine pancreatic insufficiency on nutrient digestibility, growth parameters as well as anatomical and histological morphology of the intestine in a juvenile pig model

**DOI:** 10.3389/fmed.2022.973589

**Published:** 2022-09-09

**Authors:** Dana Carina Schubert, Anne Mößeler, Bianca Ahlfänger, Marion Langeheine, Ralph Brehm, Christian Visscher, Amr Abd El-Wahab, Josef Kamphues

**Affiliations:** ^1^Institute for Animal Nutrition, University of Veterinary Medicine Hannover, Foundation, Hanover, Germany; ^2^Vetsuisse Faculty, Institute of Animal Nutrition and Dietetics, University of Zurich, Zurich, Switzerland; ^3^Institute for Anatomy, University of Veterinary Medicine Hannover, Foundation, Hanover, Germany; ^4^Department of Nutrition and Nutritional Deficiency Diseases, Faculty of Veterinary Medicine, Mansoura University, Mansoura, Egypt

**Keywords:** exocrine pancreatic insufficiency, pig model, pancreatic duct ligation, digestibility, intestinal morphology

## Abstract

In a pig model, pancreatic duct ligation (PL) leads to a complete loss of exocrine function, causing an exocrine pancreatic insufficiency (EPI) without affecting endocrine function, allowing research of clinical effects and therapy options. This study aimed to investigate effects of experimentally induced EPI in juvenile pigs on digestion and intestinal morphology. Eight female juvenile cross-bred pigs (BW 54.8 kg at the start of the study) were included. Three animals were considered as a control (CON group), and in five animals the ductus pancreaticus accessorius was ligated (PL group). During the 10-week trial period, body weight and body measurements were recorded regularly. At the end of the trial, gastrointestinal tract (GIT) was investigated macroscopically and histologically and weight and digesta samples of individual segments were obtained. The pigs in the CON showed a significantly higher apparent total tract digestibility of crude protein and crude fat (87.8 and 79.9%, respectively) compared to PL (52.4 and 16.6%, respectively). Significant differences were noted in relative weights of duodenum, jejunum and colon (with and without digesta) and also in absolute weights of jejunum and colon. The mean number of nuclei in the transverse section in stratum circulare were significantly higher in all intestinal segments in CON compared to PL. Overall, EPI results in impaired nutrient digestibility with a greater filling of the GIT with digesta. The elongation of the small intestine does not represent “stretching” of the intestine, but rather increased synthesis of intestinal tissue.

## Introduction

Exocrine pancreatic insufficiency (EPI) is a chronic illness caused by decreased synthesis and secretion of digestive enzymes of pancreatic origin (1, 2). EPI can be caused/induced by a variety of factors, including cystic fibrosis, acute or chronic pancreatitis, malignancy, and gastrointestinal surgery complications (3–5). Pancreatitis is characterized by acinar cell destruction and activation of inflammatory cells, including macrophages, neutrophils, and granulocytes, which secrete inflammatory cytokines (6).

The progression, severity, and occurrence of complications throughout the course of EPI differ significantly between patients and are related to the etiology of pre-existing diseases as well as the affected person’s lifestyle (e.g., nutrition, smoking, etc.). For EPI patients, daily problems like abdominal pain and diarrhea are common, as well as weight loss (3, 7). The symptoms have a significant negative impact on one’s quality of life, and malnutrition can cause additional serious disorders such as osteoporosis, and osteopenia (8, 9). Numerous studies have examined the association between nutritional status and lung function in cystic fibrosis patients prior to lung transplantation, and malnutrition has been identified as a key factor for worse prognosis (10). For example, vitamin D deficiency has been associated with impaired lung function in non-transplanted cystic fibrosis patients, and might be associated with an increased risk of rejection and infections after lung transplantation (11, 12). Subsequently, nutritional support has been shown to defer impairment of pulmonary function and improve survival in lung transplantation candidates (13).

In order to develop and optimize EPI therapies, *in vivo* models of the complex disease are used. The pancreatic duct ligation (PL) in pigs is a well-established technique for studying the effects of EPI on nutrient digestibility and absorption (4, 14). Adult ileo-caecally fistulated pigs are preferred as EPI models to study digestion and absorption of nutrients because of their remarkable resemblance in digestion to humans and the ease with which the isolated pancreatic duct can be ligated (15–17). In fact, the precaecal digestibility is worldwide standard to estimate protein digestibility in monogastric species. Pigs, unlike humans, have distinct bile and pancreatic ducts that flow separately into the duodenum (18). As a result, pancreatic duct ligation does not cause extrahepatic cholestasis (19).

Experimentally induced EPI in minipigs with ileocaecal fistulas were and are still used to assess digestive processes (15). In addition, the effect of pancreatic enzyme replacement therapy (PERT) was studied in PL pigs, and this kind of medication was found to improve symptoms of EPI (16). The investigation of digestive enzyme mechanisms inside the intestine is possible with fistulated EPI minipigs (16). However, malnutrition could result from the PL surgical operation because pancreatic enzyme secretion including buffering substances as well as enzymes is completely lost (17, 20, 21).

Individual young pigs with experimentally induced EPI (not treated with pancreatic enzymes) showed an elongation of the small intestine (up to 6 m without significant histological changes) within 10 wk, whereas this phenomenon was not seen in young PL pigs substituted with pancreatic enzymes (22). Prykhodko, Fedkiv (23) observed that EPI had a significant influence on the intestinal permeability of the jejunum in young pigs. Mößeler, Bergemann (24) concluded that the NBT-PABA test could be a very useful test for indirectly evaluating proteolytic enzyme efficiency *in vivo*, and also gave information about the kinetics of enzyme action [by splitting NBT-PABA to PABA; but also the fact that PABA (free) was absorbed faster/to a higher extent], which go hand in hand with findings of Prykhodko, Fedkiv (23). Mößeler, Hermann (25) found that experimentally induced EPI in pigs affects not only growth parameters or small intestinal length but also nutrient transport processes in the small intestine in a segment-specific manner. To date, information is lacking whether EPI affects the small intestinal morphology of the elongated section in young pigs. Because the effects of EPI in young pigs are thought to be age dependent (26), the current investigation involved PL young pigs at the age of 15 wk that had undergone experimentally induced EPI and were fed diets without pancreatic enzymes. Fedkiv, Rengman (26) showed that importance of the exocrine pancreatic function for growth in weaner pigs, while in older animals it played a minor role in growth. Also, Fedkiv, Rengman (26) concluded that feed supplementation with pancreatin increased the appetite and ensured an improved feed conversion. Pierzynowski, Szwiec (20) found that EPI pigs fed a regular diet exhibit extreme growth retardation compared with their normal growth curve. However, such pigs do not develop cachectic signs in long-term studies and exhibit an insufficient coefficient of fat absorption. The aim of this study was to prove previous findings of a reduced nutrient digestibility, impaired growth and elongation of the small intestine in young PL pigs. Furthermore, this study investigated which intestinal segment is in particular amenable for the elongation and how EPI affects the tunica muscularis histologically.

## Materials and methods

The trial was conducted in accordance with German regulations and approved by the Ethics Committee of Lower Saxony for the Care and Use of Laboratory Animals (LAVES: Niedersächsisches Landesamt für Verbraucherschutz und Lebensmittelsicherheit; reference: 33.9-42502-04-15/1910). In order to comply with the 3-R principle and the German animal welfare regulations, only few animals were used to study digestive processes and intestinal morphology in the PL-pig model.

### Animals, housing, and experimental design

The trial was conducted in eight female fattening cross-bred pigs (54.8 ± 4.58 kg initial BW), which were 15-wk-old at the beginning of the trial. After 1 week of acclimatization, the animals underwent laparotomy (see the section “Surgery protocol and post-operative procedure”). While three animals underwent sham OP and were only manually manipulated (control group, CON), in five animals the pancreatic duct or ductus pancreaticus accessorius was ligated (pancreas ligated group, PL) in order to experimentally induce EPI. After surgery, the animals were kept pairwise in 1 × 3 m^2^ sized pens equipped with concrete floor, nipple drinkers and rubber mats as lying area. The pairs were each composed of one CON and one PL pig, except for one pair that consisted of two PL pigs. This unequal number was chosen because, due to possible anatomical peculiarities (e.g., presence of the second excretory duct), there was a risk that no EPI could be induced by PL in one or more animals. Surgical PL in five of the eight animals was intended to ensure availability of a sufficient number of animals with EPI.

**FIGURE 1:**
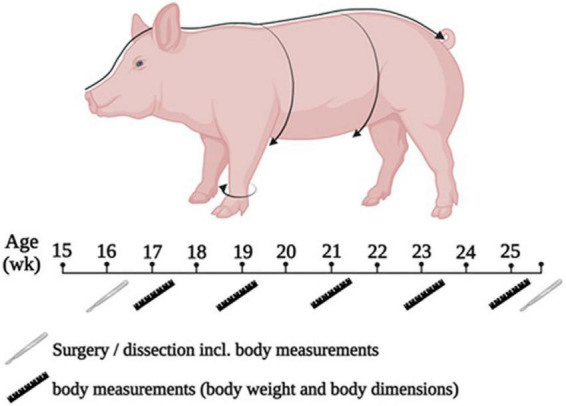
Scheme of experimental procedure and for performing the measurement of body dimensions.

The trial lasted 10 wk. During the trial, the animals were weighed once weekly after morning feeding. Every 2 wk, the circumference of the chest, abdomen, and the cannon bone, as well as the nose-rump length of each animal were determined with a measuring tape (Figure 1) while the animals underwent short anesthesia to allow precise measurements. At the end of the trial, all animals underwent dissection. On the day of the dissection, the animals were also measured and weighed.

### Surgery protocol and post-operative procedure

The animals were narcotized by means of injection with a combination of 0.023 mg ketamine hydrochloride/kg BW i.m. (Ursotamin, Serumwerk Bernburg AG, Bernburg, Germany) and 2 mg azaperone/kg BW i.m. (Stresnil, Elanco Deutschland GmbH, Bad Homburg, Germany). When the depth of anesthesia was not sufficient, a further injection was given if necessary (ketamine hydrochloride, half of the initial dose).

Ligation of the pancreatic duct (PL group) was performed according to the method described by Tabeling, Gregory (27). After preparation of the surgical field, the abdominal cavity was opened about 5 cm caudal to the xiphoid process in the linea alba for about 10 cm. The pancreatic duct was then accessed, exposed, double ligated and transected. The abdominal cavity was subsequently closed in three layers. The surgery of the control animals was similar to that of the PL animals, but the ligation of the pancreatic duct was omitted. In order to create comparable conditions to the PL animals – apart from the pancreatic duct ligation – the intestine and the pancreatic tissue were palpated manually for a few minutes.

After surgery, the animals received antibiotic treatment with 15 mg amoxicillin/kg BW i.m. (Duphamox L.A.^®^, Zoetis Deutschland GmbH, Berlin, Germany) three times at 48 h intervals and analgesic treatment with 8 mg meloxicam/kg BW/d i.m. (Metacam^®^, Boehringer Ingelheim Pharma GmbH & Co. KG, Ingelheim am Rhein, Germany) for 3 days after surgery.

One week after surgery, the chymotrypsin content in the feces was determined using the Chymotrypsin-Kit^®^ (Catalog number K6990, Immundiagnostik AG, Bensheim, Germany). The substrate Succ-Ala-Ala-Pro-Phe-pNA is converted in the presence of chymotrypsin to Succ-Ala-Ala-Pro-Phe and p-nitroaniline in the presence of chymotrypsin. This results in a color change, which is measured photometrically at 405 nm. Animals with a chymotrypsin concentration below 0.9 IU/g feces were defined as PL-animals.

### Experimental diets and feeding regime

The diet was formulated to meet or exceed nutrient requirement for growing fattening pigs (28). The animals received a complete feed that was based on wheat, barley and soybean meal (Table 1). The chemical composition of the diet is shown in Table 2. The diet was provided *ad libitum*. Fresh feed was given every morning after the feed refusals were removed. Feed refusals were weighed once weekly after drying at 103°C in order to determine the dry matter (DM) intake.

**TABLE 1:** Composition (%) of the experimental diet.

Item	Amount
Wheat	35.5
Barley	30.3
Soybean Meal	15.0
Linseed Oil	5.00
Skimmed Milk Powder	5.00
Soya Oil	3.00
Mineral Premix^1^	2.00
Brewer’s Yeast	0.56

^1^Composition (per kg premix): Calcium (220 g), phosphorus (40 g), sodium (35 g), lysine (80 g), methionine (20 g); feed additives (per kg premix): L-lysine monohydrochloride (80 g), DL-methionine (20 g), L-threonine (20 g), vitamin A (400,000 I.U.), vitamin D3 (45,000 I.U.), vitamin E (2,500 mg), iron from iron-(II)-sulfate monohydrate (4,850 mg), manganese from manganese-(II)-oxide (3,600 mg), zinc from zinc oxide (3,600 mg), copper from copper-(II)-sulfate pentahydrate (600 mg), iodine from calcium iodate anhydrous (60 mg), selenium from sodium selenite (12 mg).

**TABLE 2:** Chemical composition of the diet as analyzed.

Item	Unit	Amount
Dry matter (DM)	g/kg diet	906
Organic matter	g/kg DM	50.7
Crude rotein	g/kg DM	195
Crude at	g/kg DM	101
Crude fiber	g/kg DM	40.6
N-free extracts	g/kg DM	518
Starch	g/kg DM	402

### Dissection

In preparation for euthanasia, the animals were first anesthetized with a combination of 0.023 mg ketamine hydrochloride/kg BW i.m. (Ursotamin, Serumwerk Bernburg AG) and 2 mg azaperone/kg BW i.m. (Stresnil, Elanco Deutschland GmbH). Euthanasia was performed by intracardiac injection of T61^®^ (Intervet, Unterschleissheim, Germany; active ingredients: tetracaine hydrochloride, embutramide, mebezonium iodide; dosage: 0.24 mL/kg BW i.c.). Death of the animals was observed due to cardiac arrest, absence of the corneal reflex and dilated pupils.

The stomach and the individual intestinal sections were separated by ligation (into anatomical regions according to the macroscopical landmarks plica duodeno-colica and plica ileo-caecalis) before taking out of the abdominal cavity and then weighed with digesta (“full”) or without (“empty”) Digesta samples were obtained from each section for analysis of pH and volatile fatty acid content. The length of the intestinal sections was determined after mesentery was cut off to ensure a precise measurement. For the caecum, the width was measured in addition to the length. For histological examination, tissue samples were obtained from the duodenum, jejunum and ileum of each animal. Three samples each (beginning, middle and end of intestinal segment) were taken from the duodenum and ileum. Five samples were taken from the jejunum due to the length of this intestinal segment: at the beginning (0%), 25, 50, and 75% of the total length as well as at the end (100%) of the intestinal segment. Samples for histological examination were washed in phosphate buffered saline (PBS) and then fixed in Bouin’s solution for 48 h directly after sampling until further processing (see the section “Histological examination”).

### Determination of the nutrient digestibility

The nutrient digestibility (ND) which can be considered as apparent digestibility for protein and fat due to endogenous losses and as true digestibility for starch was calculated by means of marker method. Therefore, during the last 10 days of the trial, chromium oxide (Cr_2_O_3_) was added to the diet at a concentration of 2.51 g/kg DM. For starch, both true precaecal and true total tract digestibility were determined, and for protein and fat, apparent total tract digestibility (ATTD) was determined. For calculation, the following equation for the marker method in accordance with Kamphues (29) was applied:

$$\text{ND}\left(\%\right)=100-[\frac{\%\text{marker in diet}}{\%\text{marker in chyme}}\times \frac{\%\text{nutrient in chyme}}{\%\text{nutrient in diet}}]\times 100$$

By using the marker method, a total collection of the feces over several days can be omitted. Instead, the nutrients in the diet and in the feces are quantitatively determined and related to the respective marker concentration. To obtain the ND, in a first step the ratio of marker in diet to marker in digesta has to be multiplied with the ratio of nutrient in feces to nutrient in diet. In a second step, this result multiplied by 100 and then subtracted from 100 gives the ND in percent (%).

### Histological examination

After dissolving picric acid from the tissue by means of alcohol (70%), samples were embedded in Technovit^®^ (Technovit^®^ 8100, Haraeus Kulzer GmbH, Wehrheim, Germany) following standard protocols (30). For histological examination, 2 μm sections were stained with toluidine blue (TB). The tissue samples were evaluated using the axioscope (Carl Zeiss Jena GmbH, Jena, Germany), the associated camera and software. In the stratum circulare and stratum longitudinale of the tunica muscularis of the duodenum, jejunum and ileum, the number of nuclei was determined in 20 grid cells under 400-fold magnification using a measuring grid (grid size 40 × 40 μm).

### Analytical methods

All analyses were performed in duplicates. Feed and digesta were analyzed by standard procedures in accordance with VDLUFA (31). Dry matter was determined after drying samples at 103°C until weight constancy. Crude ash was analyzed by means of incineration in the muffle furnace at 600°C for 6 h. The DUMAS combustion method was applied to determine the total nitrogen content (Vario Max CNS^®^). Total N was multiplied by a constant factor of 6.25 to calculate the crude protein content. The ether extract was analyzed after acid hydrolysis (Ankom hydrolysis system^®^ and Ankom XT 15 Extractor). The crude fiber was determined after washing samples in diluted acids and alkalis (Fibertec 2010 Hot Extraktor^®^, Foss, Hilleroed, Denmark). The detection of starch content was performed by means of enzymatic determination (UV-method, R-Biopharm AG, Darmstadt, Germany). To analyze trace elements (Cu, Zn, Fe, Mn), atomic absorption spectrometry (Solaar M Series Atomic Absorption Spectrometer, Thermo Elemental, Cambridge, England) was used.

To determine the pH value in digesta and feces, the samples were diluted with distilled water in a ratio of 1:5 and measured after 30 min with the pH meter (pH526 Multi Cal^®^ from WTW). The determination of volatile fatty acids in the digesta and feces was carried out using gas chromatography as described by Schwarzmaier (32). In the fecal samples from the section, the Cr_2_O_3_ content was determined according to the method of Petry and Rapp (33).

### Statistical analysis

Statistical analysis was performed by means of the Statistical Analysis System for Windows SAS^®^ Enterprise Guide^®^, version 7.1 (SAS Institute Inc., Cary, NC, United States). The data are given as mean and standard deviation. The *t*-test (2-sided) was used to evaluate statistical differences between CON and PL animals. Differences were considered significant when *p* < 0.05.

## Results

### Nutrient digestibility

The results of ND along the total tract and the precaecal digestibility of starch are presented in Table 3. The pigs in the CON group showed a significantly (*p* < 0.0001) higher ATTD of crude protein and crude fat (87.8 and 79.9%, respectively) compared to PL animals (52.4 and 16.6%, respectively). However, the total tract digestibility of starch did not differ between both groups (about 99%), while there was a strong significant difference (*p* < 0.0001) in the precaecal digestibility of starch (93.4 vs. 56.0%).

**TABLE 3:** Apparent total tract digestibility (%) of protein, fat, and precaecal as well as total tract true digestibility (%) of starch of the animals in both groups (mean ± SD).

Item	CON	PL	*p*-value
Crude protein	87.8^a^ ± 1.87	52.4^b^ ± 6.19	<0.0001
Crude fat	79.9^a^ ± 0.49	16.6^b^ ± 2.22	<0.0001
Starch			
Precaecal	93.4^a^ ± 1.51	56.0^b^ ± 5.07	<0.0001
Total tract	99.0^a^ ± 0.27	99.1^a^ ± 0.38	0.7385

^a,b^Means within the same row with different superscripts differ significantly (*p* < 0.05).

### Body weight and body dimensions

During the first 2 wk of the trial (15 and 16 wk of age), there were no significant differences in BW between both groups (Table 4). Nevertheless, experimentally induced EPI (PL group) resulted in significantly (*p* = 0.002 and *p* = 0.0007) reduced BW from 17 wk up to 25 wk (63.0 and 107 kg, respectively) in comparison to CON (71.7 and 140 kg, respectively).

**TABLE 4:** Body weight (kg) of the animals in both groups (mean ± SD).

Age (wk)	CON	PL	*p*-value
15	52.0^a^ ± 6.50	56.4^a^ ± 2.56	0.2046
16	59.8^a^ ± 2.47	59.0^a^ ± 2.69	0.7152
17	71.7^a^ ± 1.53	63.0^b^ ± 2.62	0.0022
18	79.2^a^ ± 1.89	67.5^b^ ± 3.86	0.0028
19	91.8^a^ ± 2.08	74.0^b^ ± 3.82	0.0003
20	102^a^ ± 2.25	80.2^b^ ± 5.63	0.0008
21	107^a^ ± 0.764	83.8^b^ ± 6.31	0.0009
22	115^a^ ± 1.26	89.8^b^ ± 5.73	0.0004
23	124^a^ ± 2.89	95.4^b^ ± 8.45	0.0015
24	133^a^ ± 1.50	100^b^ ± 8.97	0.0008
25	140^a^ ± 1.04	107^b^ ± 8.96	0.0007
26	146^a^ ± 1.00	112^b^ ± 8.20	0.0004

^a,b^Means within the same row with different superscripts differ significantly (*p* < 0.05).

Average body measurements of animals in both groups are shown in Table 5 and Supplementary Figure 1. From 21 wk of age up to 26 weeks of age, nose-rump-length was significantly shorter in PL animals (at 26 wk: 160 vs. 151 cm) and from 17 wk of age up to 26 wk of age, pigs in the CON group had significantly higher chest circumference than in those in PL group (at 26 wk: 121 vs. 110 cm). The abdominal circumference measurements did not differ between both groups throughout the experimental period. From 19 wk and up to 26 wk of age, the cannon bone circumference of pigs in CON group showed significantly (*p* = 0.0032) higher values than those in PL group (at 26 wk: 17.7 vs. 16.7 cm).

**TABLE 5:** Average body dimensions (cm) of the animals in both groups (mean ± SD).

Age (wk)	Nose-rump length			Chest circumference			Abdominal circumference			Cannon bone circumference		
				
	CON	PL	*p*-value	CON	PL	*p*-value	CON	PL	*p*-value	CON	PL	*p*-value
16	118^a^ ± 2.65	116^a^ ± 6.78	0.6504	84.8^a^ ± 2.02	85.5^a^ ± 1.90	0.6552	96.5^a^ ± 2.65	96.6^a^ ± 2.04	0.9537	15.0^a^ ± 0.00	15.4^a^ ± 0.43	0.0925
17	124^a^ ± 3.21	125^a^ ± 7.20	0.8426	90.8^a^ ± 1.44	85.0^b^ ± 0.79	0.0003	105^a^ ± 2.02	106^a^ ± 2.95	0.6384	15.3^a^ ± 0.58	15.3^a^ ± 0.45	0.9295
19	133^a^ ± 4.16	128^a^ ± 6.73	0.3677	99.3^a^ ± 1.53	89.9^b^ ± 1.08	<0.0001	113^a^ ± 4.36	111^a^ ± 0.84	0.5499	16.0^a^ ± 0.00	15.3^b^ ± 0.45	0.0249
21	141^a^ ± 1.73	133^b^ ± 2.17	0.0014	108^a^ ± 1.53	94.8^b^ ± 3.82	0.0012	126^a^ ± 1.00	119^a^ ± 6.44	0.1200	17.0^a^ ± 0.00	16.1^b^ ± 0.22	0.0008
23	148^a^ ± 5.51	137^b^ ± 2.86	0.0083	114^a^ ± 2.08	100^b^ ± 3.87	0.0015	128^a^ ± 2.52	125^a^ ± 4.04	0.4901	17.0^a^ ± 0.00	16.2^b^ ± 0.45	0.0161
25	155^a^ ± 2.65	145^b^ ± 6.50	0.0447	120^a^ ± 0.58	104^b^ ± 5.81	0.0032	136^a^ ± 2.08	129^a^ ± 6.27	0.1153	17.5^a^ ± 0.50	16.4^b^ ± 0.42	0.0151
26	160^a^ ± 6.11	151^b^ ± 4.49	0.0426	121^a^ ± 0.58	109^b^ ± 6.18	0.0113	136^a^ ± 2.65	132^a^ ± 5.18	0.2286	17.7^a^ ± 0.29	16.7^b^ ± 0.27	0.0032

^a,b^Means within the same row with different superscripts differ significantly (*p* < 0.05).

### Relative weight and length of organs

Table 6 shows the relation of stomach and different intestinal segments masses either with or without digesta to the body mass. Relative weights of filled duodenum (0.08 vs. 0.16%), filled jejunum (1.94 vs. 4.94%) and filled colon (2.23 vs. 5.05%) were significantly increased in the PL pigs (Supplementary Figure 2). Except for the stomach (0.51 and 0.53%), all empty intestinal segments showed significant differences in their relative weight between both groups. Also, relative digesta fresh mass of jejunum (0.83 vs. 2.59%, *p* = 0.0038) and colon (1.24 vs. 2.92%, *p* = 0.0004) was significantly higher in group PL. The masses (g) of stomach as well as intestinal segments (filled or emptied) and of digesta mass (fresh) of the animals in both groups at the time of dissection (mean ± SD) were presented in details in Supplementary Table 1. Total small intestinal (SI) length was 25% higher (*p* = 0.0064) in PL pigs (2,066 vs. 2,576 cm, Table 7). A more detailed look showed that this elongation was based in jejunum (1,972 vs. 2,466 cm), while the other parts of SI did not differ between the groups.

**TABLE 6:** Relation (%) of the masses of stomach as well as intestinal segments (filled or empty) and of digesta mass (fresh) to the body weight (BW) of the animals in both groups at the time of dissection (mean ± SD).

Age (wk)	Relation of organ (filled) to BW			Relation of organ (empty) to BW			Relation of digesta mass (fresh) to BW		
	CON	PL	*p*-value	CON	PL	*p*-value	CON	PL	*p*-value
Stomach	1.83^a^ ± 0.66	2.41^a^ ± 0.90	0.3770	0.51^a^ ± 0.10	0.53^a^ ± 0.03	0.6672	1.32^a^ ± 0.56	1.88^a^ ± 0.90	0.3817
Duodenum	0.08^b^ ± 0.04	0.16^a^ ± 0.02	0.0177	0.07^b^ ± 0.02	0.11^a^ ± 0.02	0.0359	0.03^a^ ± 0.01	0.05^a^ ± 0.01	0.0851
Jejunum	1.94^b^ ± 0.30	4.95^a^ ± 0.67	0.0004	1.16^b^ ± 0.29	1.97^a^ ± 0.36	0.0170	0.83^b^ ± 0.23	2.59^a^ ± 0.62	0.0038
Ileum	0.08^a^ ± 0.03	0.14^a^ ± 0.04	0.0988	0.05^b^ ± 0.01	0.09^a^ ± 0.02	0.0259	0.03^a^ ± 0.02	0.05^a^ ± 0.03	0.3681
Caecum	0.53^a^ ± 0.08	0.82^a^ ± 0.36	0.2394	0.16^b^ ± 0.01	0.27^a^ ± 0.05	0.0123	0.37^a^ ± 0.07	0.52^a^ ± 0.38	0.5321
Colon	2.24^b^ ± 0.66	5.06^a^ ± 0.43	0.0003	1.00^b^ ± 0.099	1.90^a^ ± 0.321	0.0037	1.24^b^ ± 0.22	2.92^a^ ± 0.35	0.0004
Total GIT	6.72^b^ ± 1.60	13.5^a^ ± 1.39	0.0007	2.95^b^ ± 0.38	4.88^a^ ± 0.66	0.0039	3.82^b^ ± 1.08	8.02^a^ ± 1.57	0.0069

^a,b^Means within the same row with different superscripts differ significantly (*p* < 0.05).

**TABLE 7:** Length (cm) of the different intestinal segments of the animals in both groups at the time of dissection (mean ± SD).

Organ	CON	PL	*p*-value
Duodenum	48.3^a^ ± 7.02	56.7^a^ ± 11.1	0.2929
Jejunum	1972^b^ ± 194	2466^a^ ± 157	0.0073
Ileum	45.6^a^ ± 5.50	53.4^a^ ± 13.7	0.4106
Total small intestine	2066^b^ ± 194	2576^a^ ± 158	0.0064
Colon	537^a^ ± 2.64	562^a^ ± 37.5	0.3071

^a,b^Means within the same row with different superscripts differ significantly (*p* < 0.05).

### Digesta dry matter, pH, and fatty acids profile

**FIGURE 2:**
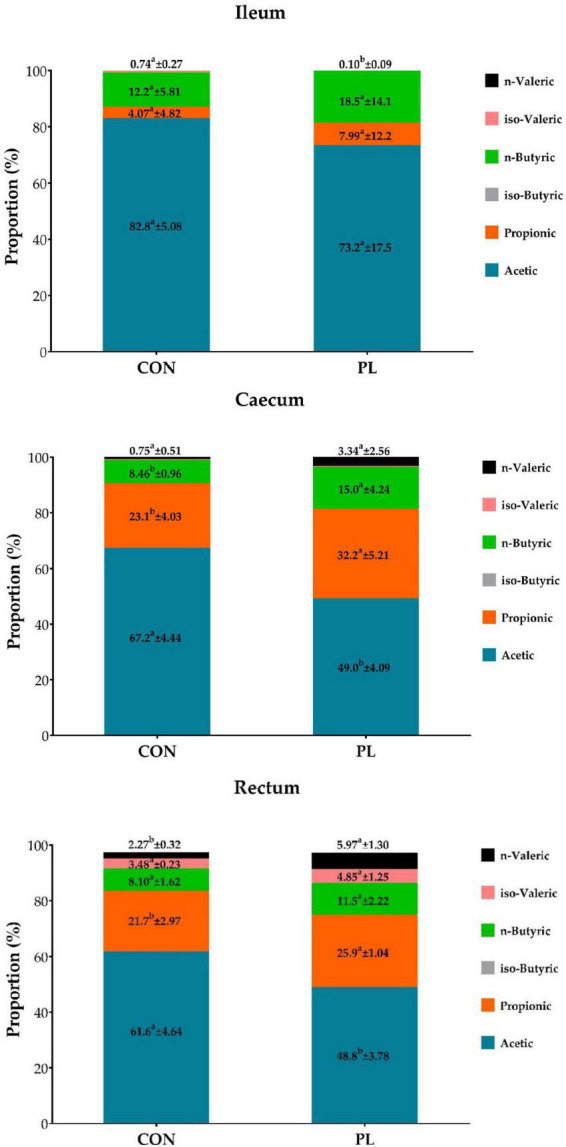
Proportion (%) of individual fatty acids in the total concentration of the fatty acids in digesta of ileum, caecum and rectum of the animals in both groups at the time of dissection (mean ± SD). ^a, b^Means within the same row with different superscripts differ significantly (*p* < 0.05).

The DM contents of the jejunum, ileum and caecum digesta were significantly higher for PL pigs while DM content in stomach, duodenum and colon were not different to CON (Table 8). Regarding digesta pH, significantly lower values were observed in ileal (6.45 vs. 5.57, *p* = 0.0389) and jejunal digesta (6.14 vs. 5.79, *p* = 0.2924) of PL animals. Significant differences were noted in the fatty acids profile of the jejunum, ileum and caecum digesta of pigs in CON and PL groups (Figure 2).

**TABLE 8:** Dry matter content (g/kg FM^1^) and the pH values of the digesta in the individual sections of the GIT of the animals in both groups at the time of dissection (mean ± SD).

Organ	DM			pH		
	CON	PL	*p*-value	CON	PL	*p*-value
Stomach	308^a^ ± 11.0	286^a^ ± 56.0	0.5285	4.38^a^ ± 0.10	4.23^a^ ± 0.43	0.5761
Duodenum	128^a^ ± 29.3	115^a^ ± 31.3	0.6045	6.45^a^ ± 0.11	6.53^a^ ± 0.52	0.8027
Jejunum	162^b^ ± 4.16	256^a^ ± 19.9	0.0002	6.14^a^ ± 0.10	5.79^b^ ± 0.50	0.2924
Ileum	129^b^ ± 16.5	226^a^ ± 42.7	0.0144	6.45^a^ ± 0.07	5.57^b^ ± 0.50	0.0389
Caecum	118^b^ ± 21.4	156^a^ ± 20.6	0.0475	5.68^a^ ± 0.25	5.54^a^ ± 0.20	0.4099
Colon	213^a^ ± 20.0	238^a^ ± 12.4	0.0703	6.27^a^ ± 0.27	6.19^a^ ± 0.30	0.7269

^1^FM, fresh matter. ^a,b^Means within the same row with different superscripts differ significantly (p < 0.05).

### Histopathological findings

**FIGURE 3:**
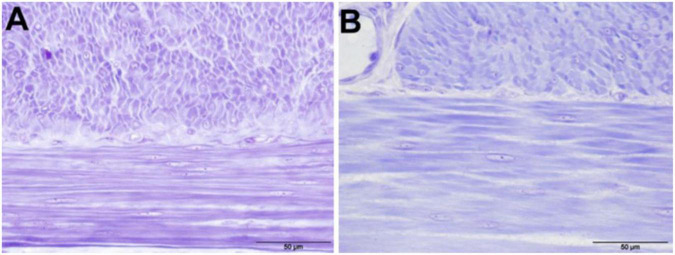
Cell nuclei in the tunica muscularis of the jejunum of animals in both groups, 400 × magnification (toluidine blue stained) **(A)** CON; **(B)** PL.

The mean number of nuclei in the stratum circulare showed significantly higher numbers in all intestinal segments for pigs in CON group compared to those in PL group (Figure 3 and Table 9). In the stratum longitudinale, significant differences were observed both in jejunum (1.2 vs. 0.67 nuclei/cell 40 μm × 40 μm) and ileum (0.90 vs. 0.71 nuclei/cell 40 μm × 40 μm) of pigs in CON group compared to those in PL group.

**TABLE 9:** Mean number of nuclei (n/cell 40 μm × 40 μm) in the stratum circulare (transvers section) and stratum longitudinale (longitudinal section) of the tunica muscularis of the animals in both groups (mean ± SD).

Item	Stratum circulare (transvers section)		Stratum longitudinale (longitudinal section)	
	CON	PL	CON	PL
Duodenum	4.68^a^ ± 2.28	2.99^b^ ± 1.73	0.95^a^ ± 0.90	0.90^a^ ± 0.95
Jejunum	3.71^a^ ± 2.17	2.20^b^ ± 1.53	1.20^a^ ± 1.18	0.67^b^ ± 0.73
Ileum	3.20^a^ ± 1.83	2.65^b^ ± 1.51	0.90^a^ ± 0.88	0.71^b^ ± 0.77

^a,b^Means within the same row with different superscripts differ significantly (*p* < 0.05).

## Discussion

Exocrine pancreatic insufficiency is a serious disease in humans and several mammalian species that, in addition to physical discomfort, is in humans also associated with stress and shame due to flatulence and diarrhea (34). Due to its anatomical and physiological similarity to the human digestive tract, the pig can serve as an *in vivo* model for the study of EPI (35–37). The great advantage of this model is that the ligation of the pancreatic duct (ductus pancreaticus accessories) results in a complete loss of exocrine function, while endocrine function is not impaired (in contrast to other species) (19). The aim of the present study was to evaluate effects of experimentally induced EPI on growth and nutrient digestion as well as on anatomical and histological properties of the intestines in 16 to 26 wk-old fattening pigs.

Results from the present study revealed that apparent crude protein digestibility (87.8 vs. 52.4%) and crude fat digestibility (79.9 vs. 16.6%) were markedly reduced in the PL animals, while starch digestion along the total tract remained unaffected. Similar results were obtained by Mößeler, Schwarzmaier (38), who found a protein digestibility of 85.0 and 49.5%, respectively, and fat digestibility of 80.1 and 18.4% in control and PL pigs, respectively. Furthermore, these findings are in agreement with studies in humans affected by EPI (39, 40). In general, in juvenile pigs, apparent crude protein digestibility could be far less important and far less effective for growth than in neonate pigs. The fact that the large intestine can compensate the lack of digestive capacity of the small intestine by microbial fermentation of starch (15, 36, 41) explains why neither this nor other studies have demonstrated an effect of EPI on total tract starch digestion in the pig model (27, 38, 42). This is also the reason why starch digestion is often not considered as critical in human patients – but many studies in ileocaecal fistulated pigs and investigations on precaecal starch digestibility in PL-pigs showed the severe reduction of precaecal enzymatic starch digestion and the massive fermentative starch digestion, which was also demonstrated in the present study by a marked reduction of precaecal starch digestibility in the PL pigs compared to CON pigs (93.4 vs. 56.0%). The fermentation of starch is one major factor causing meteorism and flatulence as well as diarrhea in EPI patients – impairing quality of life (43–45).

Although CON and PL animals did not differ in terms of BW and body dimensions at the beginning of the trial, BW as well as chest and cannon bone circumference were significantly lower 1 wk (BW, chest circumference) and 3 wk (cannon bone) after surgery, respectively, in group PL. This finding can be seen in accordance with reduced nutrient digestibility as less digestible energy and nutrients were available to the PL animals. Feed intake cannot be compared between the groups as the animals were kept pairwise. However, as one pair consisted of two PL animals, the feed intake of this pair was compared with the feed intake of the other three (mixed) pairs and no significant difference was found (*p* = 0.307, data not shown). Furthermore, no difference in feed intake between control and PL pigs could be found in previous studies (38). A reduced growth in young individuals are common findings in EPI patients (25) and are also reported for humans as well as other mammalian species, e.g., dogs (46–48). In contrast to the present study, which found only a retardation in growth, Gregory, Tabeling (49) reported a weight loss in 12-wk-old German Landrace pigs for the following 10 weeks after pancreatic duct ligation. In agreement with Mößeler, Schwarzmaier (38), in the present study body length (nose to tail) was significantly shorter in the PL animals (wk 26: 160 vs. 151 cm), and both studies found no effect on abdominal girth due to EPI (38). It can be assumed that the larger amount of digesta in the GIT in the PL animals (Table 6) led to the same abdominal girth despite their smaller body size. Furthermore, the marked increase of total digesta mass (3.82% of BW vs. 8.02% of BW, *p* = 0.0069) is of practical relevance as this might lead to an overestimation of body condition in EPI patients (38).

The entire small intestine was significantly longer in the PL animals than in the control animals (20.7 vs. 25.8 m). However, the measurement of the single intestinal segments showed that this elongation was basically due to a marked lengthening of the jejunum by 25% (19.7 vs. 24.7 m). An elongation of the small intestine in EPI pigs was previously reported (25, 50), without focusing on separate intestinal segments. Furthermore, in the present study the colon of the PL animals was significantly heavier compared to CON both, filled with digesta (3.28 vs. 5.63 kg) and empty (1.45 vs. 2.11 kg; Supplementary Table 1), respectively. On the one hand, the jejunum, with its long villi and thus large absorption surface and many transport proteins, represents the main site for nutrient breakdown and absorption (51, 52), and on the other hand, inside the large intestine, microbial fermentation (of precaecally undigested nutrients, especially carbohydrates) takes place (53, 54). Due to EPI, there is an increased flux of undigested starch into the colon, which results in an enhanced fermentation of the starch (15, 55). Therefore, an enlargement of these intestinal segments (jejunum and colon) seems most effective in compensating for the decreased digestive capacity in EPI patients.

In order to clarify whether the lengthening of the small intestine was merely due to a stretching of the small intestinal tube or an increase in mass, the absolute intestine weight and the relation of intestine length to intestine weight were determined. The result of the latter was approx. 90 g/m in both groups, which indicates that the intestinal wall thickness did not differ between the groups and that an actual growth of the intestine in the PL animals can be assumed. Previous studies found by histological or ultrasonographic examination either no effect on intestinal wall thickness due to EPI or an increase in intestinal wall thickness in EPI patients (56–59).

To further clarify which mechanisms underlay the elongation of the intestine at the cellular level, small intestine samples were examined histologically by means of microscopic nuclei count in the tunica muscularis. The number of nuclei (per 40 μm × 40 μm cell) in the stratum circulare (transvers section) was significantly lower in all intestinal segments in the PL compared to CON animals. From this, a lower number of smooth muscle cells per defined area in the tunica muscularis of PL pigs can be deduced. The growth in length of the intestine could thus be due to hypertrophy of the smooth muscle cells rather than increased cell division. This in agreement with Lærke and Hedemann (60) who found that in pigs the small intestines grows most intensively within the first 3–4 months of life and also with previous studies that found effects of experimentally induced EPI depend on the age of the pigs at which they underwent PL surgery (23, 25, 26). However, from the results of the present study it can be deduced that the intestine, which has (nearly) completed its physiological growth, can still react in case of a loss of digestive capacity. Similar observations have already been made in (adult) humans who have had part of their small intestine surgically removed which results in a state called short bowel syndrome (SBS) (61, 62). Also in mice and rats, an elongation of the small intestine was observed after experimentally induced SBS (63, 64). Comparable to EPI, this shortening of the intestine is also associated with an increased influx of precaecal undigested nutrients into the large intestine and led to an increase in the length of the small intestine after surgery (52, 65).

Dry matter content was increased in the digesta of jejunum, ileum, and caecum of the PL animals. This finding goes in line with Tabeling et al. (27), who also found that DM content in ileal digesta was higher in fistulated PL minipigs compared to control minipigs (144 vs. 213 g/kg) and linked this result to the lack of dilution by the pancreatic juice. A reduction in pH was found in jejunal (6.14 vs. 5.79) and ileal digesta (6.45 vs. 5.57), but not in other segments of the GIT. In contrast, Gregory, Tabeling (49) reported a marked reduction of duodenal pH by 2.0 units in German Landrace pigs (Con: 6.81 vs PL: 4.79). Tabeling, Gregory (27) also found a significant reduction of pH in ileal digesta of PL compared to control minipigs (7.74 vs. 7.33), but do not provide results of other GIT sections. Gregory, Tabeling (49) assumed that the reason for the acidic pH was that the buffering effect of the pancreatic bicarbonate secretion was missing. However, the buffering effect of the pancreatic juice is limited and, furthermore, greatest pre-prandial (27), but dissection in the present study was performed post-prandial. A typical finding in EPI patients is a state called small intestinal bacterial overgrowth (SIBO), which results due to increased nutrient availability in the aboral part of the small intestine (49, 66, 67). In addition, the pancreatic juice is said to have an antibacterial effect and its absence further favors bacterial colonization (68, 69). As a consequence, short-chain fatty acids (SCFA) can be formed by microbial fermentation which reduce the intestinal pH-value (43). In the present study, the concentration of SCFA in ileal digesta was 73.0 mmoL/g FM in the PL and 38.8 mmoL/g FM in the CON animals (data not shown). Although this difference was not significant, it might have contributed to the decreased pH of ileal digesta in the PL pigs. Regarding the profile of SCFAs, a clear shift in the proportions could be shown especially in caecal digesta and less pronounced in rectal digesta, whereby a lower proportion of acetic acid with a concomitantly higher proportion of propionic and butyric acid was observed in the PL animals. Butyric acid is reported to have an epitheliotrophic effect. Therefore, changes in SCFA profile might have contributed to the increased organ weight of the colon. Previous studies have found a correlation between increased nutrient concentrations in the distal ileum and serum levels of the growth factor GLP-2, so that the authors suggested that this was the cause of the increased intestinal growth (50). The role of dietary fat of 5% can be minor in juvenile pigs and EPI in general. The SCFA of 5% dietary fat can be almost neglected when the human diet is 20–30% fat and even higher in people with cystic fibrosis 30–40%. Thus, we recommend in next future studies to participate pigs with BW of about 110 kg and to use diet with 20–30% fat which can mimic human diet.

## Conclusion

Exocrine pancreatic insufficiency results in reduced apparent total tract digestion of crude protein and crude fat. This is on the one hand associated with a reduced growth of the PL animals and on the other hand with a greater filling of the GIT with digesta, mainly in the jejunum, ileum and colon. Based on these results, an increased influx/flooding of unabsorbed nutrients from the small into the large intestine can be deduced. Furthermore, it has been shown that EPI induces an elongation of the jejunum. In relation to the length of the small intestine, the empty masses of the small intestine did not differ between the animals of the PL group and the animals of the control group. Therefore, elongation of the small intestine does not represent “stretching” of the intestine, but rather increased synthesis of intestinal tissue. More specifically, there is a hypertrophy of the smooth muscle cells in the tunica muscularis (in PL animals).

## Data availability statement

The original contributions presented in this study are included in the article/Supplementary material, further inquiries can be directed to the corresponding author.

## Ethics statement

The animal study was reviewed and approved by the Ethics Committee of Lower Saxony for the Care and Use of Laboratory Animals (LAVES: Niedersächsisches Landesamt für Verbraucherschutz und Lebensmittelsicherheit; reference: 33.9-42502-04-15/1910).

## Author contributions

DS, AM, BA, and AAE-W: data collection. ML, RB, and JK: study design. DS and BA: statistical analyses. DS, AAE-W, and CV: writing. All authors have read and approved the manuscript.
