# Supplementary material for: Influences of exocrine pancreatic insufficiency on nutrient digestibility, growth parameters as well as anatomical and histological morphology of the intestine in a juvenile pig model
Source: Front Med (Lausanne). 2022 Sep 9;9:973589. doi: 10.3389/fmed.2022.973589 (PMC9505903; doi:10.3389/fmed.2022.973589)
Supplement: Supplementary file 1 [file Data_Sheet_1.pdf]

Supplementary Materials

Table S1. Masses (g) of stomach as well as intestinal segments (filled or emptied) and of digesta mass (fresh) of the animals in both groups at the time of dissection (mean ± SD).

Item	Organ filled		Organ emptied		Digesta mass (fresh)	
	CON	PL	CON	PL	CON	PL
Stomach	2678[a] ± 962	2706[a] ± 1079	747[a] ± 150	595[a] ± 62.7	1932[a] ± 812	2111[a] ± 1072
Duodenum	119[a] ± 69.3	176[a] ± 27.5	108[a] ± 34.3	123[a] ± 18.8	39.4[a] ± 20.2	52.9[a] ± 15.7
Jejunum	2837[a] ± 432	5529[b] ± 885	1703[a] ± 428	2183[a] ± 288	1217[a] ± 336	2911[b] ± 830
Ileum	122[a] ± 38.8	153[a] ± 46.6	67.7[a] ± 16.1	100[a] ± 23.2	42.7[a] ± 27.6	65.1[a] ± 5.82
Colon	3275[a] ± 969	5633[b] ± 441	1458[a] ± 145	2114[b] ± 330	1810[a] ± 321	3254[b] ± 368

[a, b] Different superscript indicate significant differences between CON and PL ($p < 0.05$). Means with the same superscript are not significantly different.

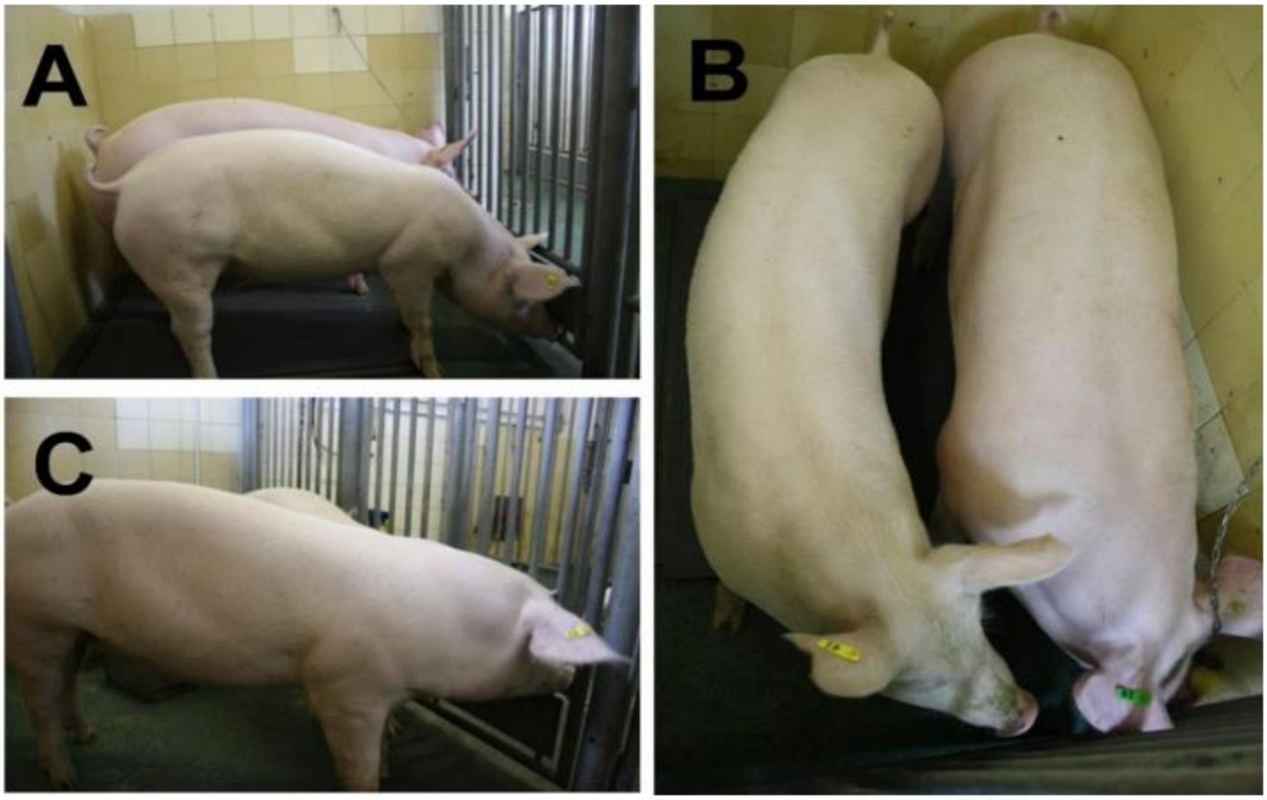

Figure S1. Control (CON) and pancreatic duct ligated (PL) animal. A: PL animal in front, CON animal in back. B: PL animal left, CON animal right. C: PL animal

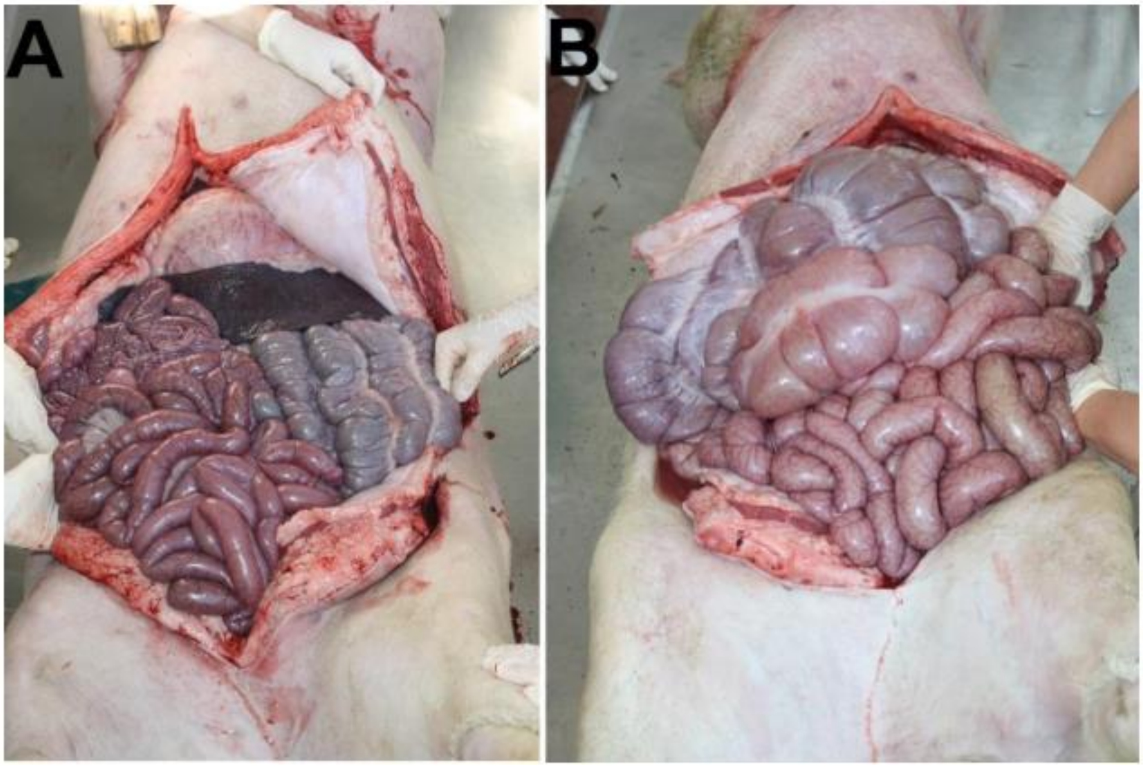

Figure S2. Abdominal cavity situs. A: Control (CON) animal. B: Pancreatic duct ligated (PL) animal.
